# Characterization of the complete chloroplast genome of *Apocynum venetum* L. (Apocynaceae)

**DOI:** 10.1080/23802359.2020.1780171

**Published:** 2020-06-16

**Authors:** Mary Ann C. Bautista, Zhongqi Xiao, Yan Zheng, Shenyu Miao, Yunfei Deng, Tao Chen

**Affiliations:** a Key Laboratory of Plant Resources Conservation & Sustainable Utilization, South China Botanical Garden, Chinese Academy of Sciences, Guangzhou, China; b Key Laboratory of South Subtropical Plant Diversity, Shenzhen Fairy Lake Botanical Garden, Chinese Academy of Sciences, Shenzhen, China; c University of Chinese Academy of Sciences, Beijing, China; d Forestry and Grassland Administration, Xinjiang Uygur Autonomous Region, Urumqi, China; e School of Life Sciences, Guangzhou University, Guangzhou, China

**Keywords:** *Apocynum venetum*, chloroplast genome, Apocynaceae, phylogenetic analysis

## Abstract

*Apocynum venetum* L. (Apocynaceae) or Luobuma is a widely known traditional medicine use to treat hypertension, relieve anxiety, soothe the nerves and promote diuresis. In this study, the complete chloroplast genome of this medicinal plant was determined through Illumina sequencing method. The *A. venetum* cp genome is 150,897 bp in length, containing a small single copy region (17,256 bp), a large single copy region (81,957 bp), and a pair of IR regions (25,842 bp). It encodes for a total of 131 genes, including 86 protein-coding genes, 8 rRNA genes, and 37 tRNA genes. Phylogenetic analysis also reveals that *A. venetum* is relatively close to *Aganosma cymosa.*

*Apocynum venetum* L. (Apocynaceae), commonly known as Luobuma, is a traditional Uygur medicine popular in Xinjiang, China (Xie et al. 201 2). Various ethnopharmacological researches documented that *A. venetum* can successfully lower blood pressure and has antihepatotoxic, cardiotonic, anxiolytic and antioxidant activities (Xie et al. 2012). Luobuma tea has also been commercially recognized as an anti-hypertensive, anti-platelet aggregation, anti-depressant, anti-aging, and sedative nutritional supplement thus gaining its popularity in China, Japan and American health food markets (Xie et al. 2012; Gao et al. 2019). But despite its widely held medicinal value, genomic information about this plant is still lacking. Closely related herb *Apocynum pictum* Schrenk is morphologically similar and easily confused for *A. venetum*, which can possibly cause misconception and problems in medicine and health product markets (Xie et al. 2012). Hence, this report presents the complete chloroplast genome sequence of *A. venetum* to provide basic genetic resource that can be used in authenticating this highly medicinal plant species.

Genomic DNA was extracted using the CTAB method from five grams of *Apocynum venetum* fresh leaves collected in Keziertuoan, Jinghe County, Bortala Mongol Autonomous Prefecture, Xinjiang, China (N44.4440; E82.1680). Voucher specimen (Chen et al. 2019062401) was deposited in the herbarium of Sun Yat-sen (Zhongshan) University (SYS). The genome sequencing was performed in Illumina Hiseq 2500 platform producing 150 bp paired end reads. High-quality reads were assembled into contigs via the de novo assembler SPAdes 3.11.0, using a k-mer set of 93, 105, 117, 121 (Nurk et al. 2013). The *A. venetum* cp genome protein coding genes annotation was conducted using CpGAVAS (Liu et al. 2012) while tRNA genes were annotated in tRNAscan-SE v2.0 (Chan and Lowe 2019).

The complete chloroplast genome of *A. venetum* (GenBank accession number: MT313688) exhibits a quadripartite structure with a length of 150,897 bp, including a large single copy (LSC) region (81,957 bp), a small single copy (SSC) region (17,256 bp) and two inverted repeats (IR) regions (25,842 bp). Overall GC content is 38.3%. A total of 131 genes were determined, including 86 protein-coding genes, 8 rRNA genes, and 37 tRNA genes. This accounts for the 61% of the whole *A. venetum* cp genome. Similar to other angiosperm cp genomes, *A. venetum* cp genome contains genes with introns. The genes *trnK-UUU, rps16, trnG-UCC, atpF, rpoC1, trnL-UAA, trnV-UAC, petB, petD, rpl16, rpl2, ndhB, trnI-GAU, trnA-UGC, ndhA* have one intron each while *clpP, ycf3* contain two introns. Trans-splicing event was also observed in *rps12* gene.

Phylogenetic analysis was carried out using RAxML 8.2.11 with the GTRI + G nucleotide substitution model (Stamatakis 2014). Protein coding regions of *A. venetum* cp genome and 19 other reported Apocynaceae cp genome sequences were used to reconstruct the phylogenetic tree. Analysis reveals that *A. venetum* is closely-related to *Aganosma cymosa* and both belong to tribe Apocyneae (Figure 1). However, some relative positions did not support the traditional classification of Apocynaceae at the subfamily level. Most placements are consistent to the published phylogenetic tree presented by Rao et al. (2019).

**Figure 1.**
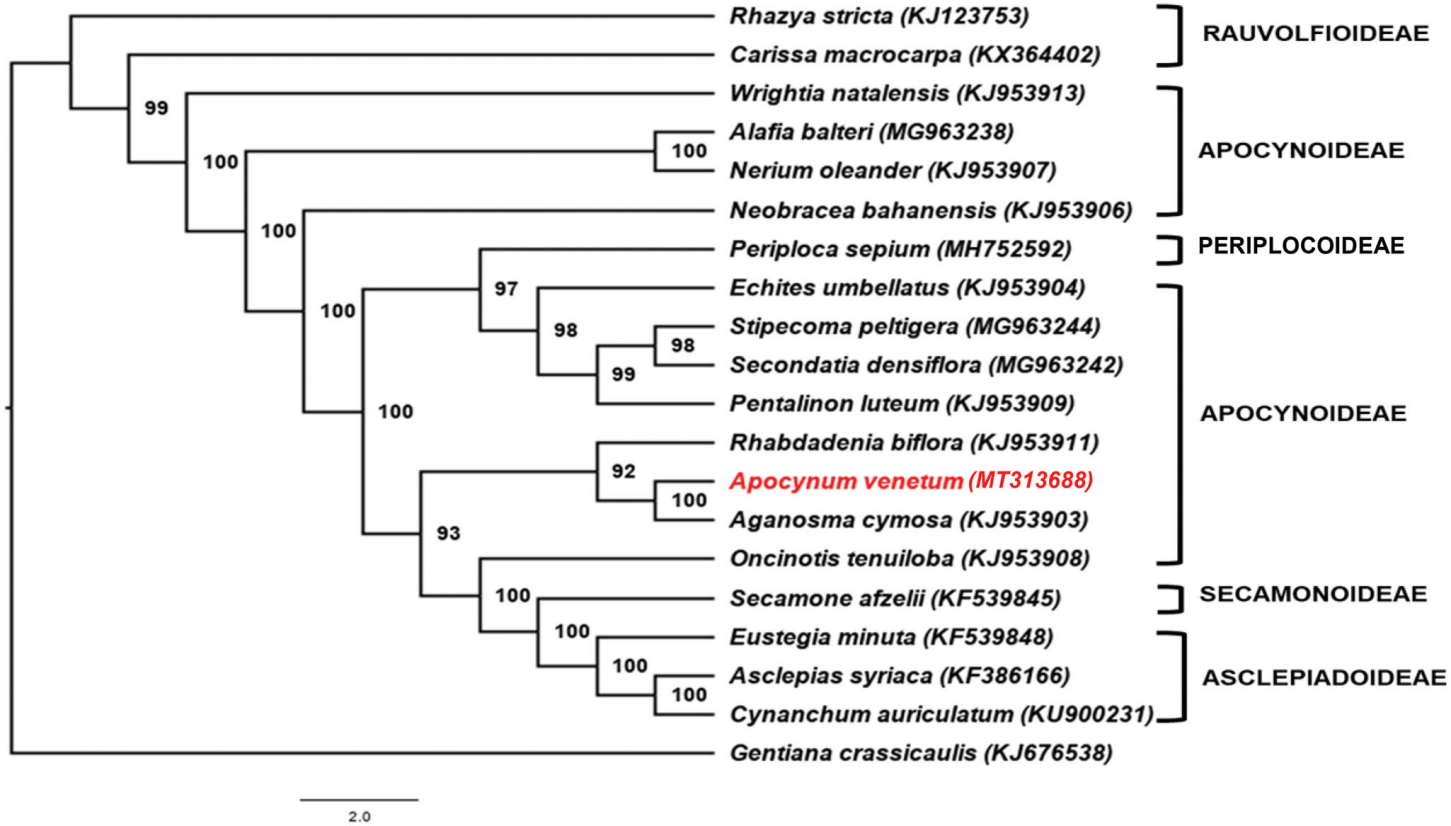
Maximum likelihood (ML) tree reconstruction of 19 taxa in Apocynaceae based on 72 shared CDS in the chloroplast genomes. Numbers on each node are bootstrap support values. *Gentiana crassicaulis* was used as the outgroup.

## Data Availability

All the chloroplast genome sequences used in this research are available in GenBank database. The complete mitochondrial genome of *Apocynum venetum* is deposited in NCBI GenBank with the accession number MT313688. (https://www.ncbi.nlm.nih.gov/nuccore/MT313688)
